# Risk factors and prognosis of pain events during mechanical ventilation: a retrospective study

**DOI:** 10.1186/s40560-017-0212-5

**Published:** 2017-02-08

**Authors:** Ayahiro Yamashita, Masaki Yamasaki, Hiroki Matsuyama, Fumimasa Amaya

**Affiliations:** 1 Department of Anesthesiology, Kyoto Prefectural University of Medicine, Kajiicho 465, Kamigyo-Ku, Kyoto 602-8566 Japan; 2 Department of Anesthesia, Japanese Red Cross Kyoto Daiichi Hospital, Kyoto, Japan

**Keywords:** Behavioral pain scale, Mechanical ventilation, Risk factors, Prognosis

## Abstract

**Background:**

Pain assessment is highly recommended in patients receiving mechanical ventilation. However, pain intensity and its impact on outcomes in these patients remain obscure. We collected the results of routine pain assessments, utilizing the behavioral pain scale (BPS), from 151 patients receiving mechanical ventilation. Risk factors associated with a pain event, defined as BPS of >5, and its impact on patient outcomes were investigated.

**Methods:**

A total of 151 consecutive adult patients receiving mechanical ventilation for more than 24 h in a single 10-bed ICU were enrolled in this study. The highest BPS within 48 h after the initiation of mechanical ventilation was collected, as well as information about the patients’ characteristics and medication received. We also recorded patient outcomes, including time to successful weaning from mechanical ventilation, time to successful ICU discharge, and 30-day in-hospital mortality. Multivariate logistic regression analysis was used to determine factors independently associated with patients with a BPS of >5. Clinical outcomes were also assessed using multivariate logistic regression analysis, correcting for risk factors.

**Results:**

We analyzed 151 patients. The median highest BPS was 4. The percentage of patients who recorded a BPS of >5 was 19.9% (*n* = 30). Multivariate logistic regression analysis revealed that the disuse of fentanyl and inotropic support was an independent predictor of pain event. Multivariable Cox regression analysis suggested that the development of a BPS of >5 was associated with increased mortality and a not statistically significant trend towards prolonged mechanical ventilation.

**Conclusions:**

A significant proportion of ventilated patients experienced a BPS of >5 soon after the initiation of mechanical ventilation. Disuse of fentanyl and use of inotropic agents increased the risk of developing a BPS of >5 during mechanical ventilation. An association between adequate analgesia and improved patient outcomes provides a rationale for the assessment of pain during mechanical ventilation, with subsequent intervention if necessary.

Pain events were common among ventilated patients. In critical care settings, appropriate and adequate pain management is warranted, given the association with improved patient outcomes.

## Background

In the critical care setting, routine pain assessment is associated with a decreased use of sedative agents, reduced duration of mechanical ventilation, a lower risk of nosocomial infection and reduced ICU stay [1, 2]. Clinical practice guidelines for the management of pain, agitation, and delirium in adult patients in the ICU (PAD guidelines) [3] recommend the implementation of pain assessment in the intensive care setting. The behavioral pain score (BPS) has been developed to measure the intensity of pain in patients receiving mechanical ventilation [4]. Reliability of the BPS has been demonstrated by the observation of increased scores during painful procedures [5–7].

Pain is considered to be common among critically ill patients [2, 8]. A previous study reported that 40% of ICU patients experienced “pain” defined as a BPS of >5, whereas 16% of ICU patients experienced “severe pain” defined as a BPS of >7 [1]. While there have been several attempts to develop pain treatment algorithms based on the BPS [9], the impact of an elevated BPS on patient outcomes remains unclear.

We hypothesized that pain event occurs within a distinctive subpopulation of patients during mechanical ventilation, and that this event is associated with poor clinical outcomes. To test this hypothesis, we retrospectively analyzed the results of routine BPS measurements during the first 48 h after the initiation of mechanical ventilation as well as patient characteristics. Based on these data, we identified risk factors for increased BPS condition during mechanical ventilation. In addition, we found an association between increased BPS and poor patient outcomes including increased mortality rate, increased duration of mechanical ventilation, and increased duration of ICU stay.

## Methods

This retrospective study was conducted in a 10-bed general ICU of a tertiary referral hospital. The study enrolled consecutive adult patients who received mechanical ventilation for more than 24 h in the ICU between September 2012 and June 2013. Patients were excluded if they were younger than 16 years old, had severe brain injury or quadriplegia, were in a deep coma before the mechanical ventilation, received surgery during the observational period, were treated with muscle relaxants, received noninvasive mechanical ventilation, or if there was any missing data in the patients’ records. Approval for data collection was obtained from the hospital’s institutional review board.

### ICU management

The patients were sedated with propofol, midazolam, or dexmedetomidine to achieve a Richmond agitation-sedation scale (RASS) score of 0 to −2. The intensivist in charge determined the target RASS level and selected the appropriate sedative regimen for each patient. Infusion rates were regulated by attending nursing staff, based on the observed RASS level. Fentanyl was used to maintain adequate analgesic condition. Adequate analgesic condition was determined by attending nursing staff. Agents were continuously infused intravenously. Protocoled regimens for sedatives/analgesics were determined for each patient. The BPS was recorded every 2–4 h in each patient who received mechanical ventilation. The score was evaluated by attending nurses trained in the use of the BPS. The BPS was evaluated when patients did not undergo any ICU related-procedure, such as tracheal suctioning or mobilization.

Mechanical ventilation was performed utilizing pressure-controlled, synchronized intermittent mandatory ventilation (SIMV) and/or pressure support ventilation (PSV). The fraction of inspired oxygen (FiO_2_), level of positive end-expiratory pressure (PEEP), and respiratory rate were adjusted to maintain arterial oxygen partial pressure (PaO_2_) between 80 and 120 mmHg and arterial carbon dioxide partial pressure (PaCO_2_) between 35 and 50 mmHg. The decision to extubate was made after a trial of spontaneous breathing with low-level pressure support ventilation (7 cmH_2_O or less). Hemodynamic management was tailored according to the patient’s clinical status, including appropriate volume expansion therapy and treatment with inotropes and/or vasopressors.

### Data collection

The highest BPS within 48 h after initiation of mechanical ventilation was recorded. Data collected included age, gender, body weight, height, surgery (cardiac or non-cardiac), and the acute physiology and chronic health evaluation II (APACHE II) score at admission. In addition, we recorded systolic blood pressure, P/F ratio as well as the use of fentanyl, propofol, dexmedetomidine, midazolam, and any kind of inotropic agents at the time of the highest BPS. Information regarding time to successful weaning from mechanical ventilation, time to successful ICU discharge, and 30-day in-hospital mortality was also collected.

Patients were divided into two groups: a pain event group in which the highest BPS exceeded 5 and a control group in which the highest BPS was 5 or under. A BPS of >5 was determined based on a previous study that defined a BPS of >5 as a “pain event” [1], and the description of a BPS of >5 as an “inadequate state” in the PAD guidelines [3]. In patients with a BPS of >5, the duration for which the BPS was >5 was collected.

The primary aim of our investigation was to determine the frequency and risk factors associated with a BPS of >5. Based on a previous study, we estimated that 40% of our patients would experience a BPS of >5. To ensure an adequate logistic regression analysis for the 6 explanatory variables, we considered that 60 observations would be required. We therefore selected 150 patients as our overall sample size.

#### Risk factor assessment

Univariate logistic regression analysis was used to identify parameters associated with pain events in the pain event group. Normality was checked using the Kolmogorov-Smirnov test, while homogeneity of variance was checked by *F* test. Student’s *t* test, the Welch test, or the Wilcoxon rank-sum test was used for continuous data as appropriate. Chi-square or Fisher’s exact test were used for categorical variables. Thereafter, a multivariate logistic regression analysis was used to determine factors independently associated with pain events in the pain event group. Variables were entered into a model when they were associated with pain status. This was based on a univariate logistic regression analysis significance threshold of *p* < 0.1, and when there was no mutual correlation, based on a Spearman’s correlation coefficient more than 0.7 or less than −0.7. The final model was constructed utilizing backward elimination of non-significant variables. Odds ratios and 95% confidence intervals (95%CI) were calculated based on the likelihood ratio statistic.

#### Clinical outcome assessment

A comparison of the three components of the BPS was performed using Friedman’s test followed by Dunn’s multiple comparison test. Univariate analysis of clinical outcomes was performed for the time to successful weaning from mechanical ventilation, time to ICU discharge, as well as 30-day in-hospital mortality rate using the log-rank test. In addition to these univariate analyses, clinical outcomes were assessed using multivariate logistic regression analysis, correcting for risk factors that showed at least a trend toward significance (*p* < 0.1) in the univariate analysis. Parameters were checked for linearity, and nonlinear parameters were entered into the model as nominal variables. Odds ratios as well as 95%CI were calculated for the outcomes. All values resulted from two-sided statistical tests, and a *p* value ≤ 0.05 was considered to be significant. R statistics (R, version 2.15.2) were used to analyze data.

Categorical data was expressed as a number (percentage). Continuous data was expressed with reference to the mean ± standard deviation (SD) or median (IQR), as appropriate.

## Results

During the study period, a total of 177 patients admitted to the ICU received mechanical ventilation for more than 24 h. Twenty-six patients were excluded from the study based on the defined exclusion criteria. We therefore analyzed 151 patients. The mean patient age was 68.5 ± 12.9 years. Overall, 66.9% were male. The median APACHE II score on admission was 19 (5–48). Fentanyl was used in 104 (68.9%) patients. All patients received at least one of the following sedatives: propofol [*n* = 89 (59.6%)], dexmedetomidine [*n* = 46 (30.5%)], and midazolam [*n* = 18 (11.9%)]. Half of the patients received inotropic support [*n* = 75 (49.7%)].

### Incidence and predictors of a BPS of >5 during mechanical ventilation

**Fig. 1:**
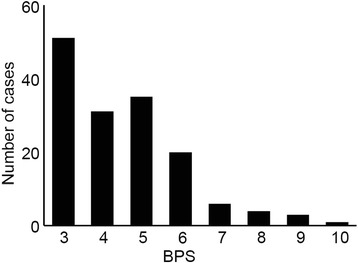
Distribution of the highest BPS within 48 h after the initiation of mechanical ventilation

**Fig. 2:**
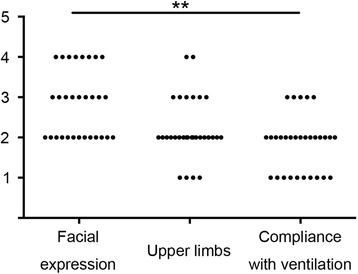
Distribution of the three components of BPS during the period in which the BPS was >5. The score for “facial expression” was significantly higher than that for “compliance with ventilation”. **: *p* < 0.01

Figure 1 demonstrates the distribution of the highest BPS within 48 hours after the initiation of mechanical ventilation. The median highest BPS was 4.0 (range, 3.0–5.0). The overall incidence of patients who experienced a BPS of >5 was 19.9% (*n* = 30). The highest BPS was recorded on day 1 in 12.6% (*n* = 19) and on day 2 in 7.3% (*n* = 11) of patients. The median duration of the period in which the BPS was >5 was 2.0 h (range, 1.0–2.3 h). In more than 95% of patients, the BPS value declined to 5 or less at the next BPS measurement. The distribution of the three components of the BPS during the period in which the BPS was >5 is shown in Fig. 2. The score for “facial expression” was significantly higher than that for “compliance with ventilation”. The RASS values recorded during the period in which the BPS was >5 are shown in Table 1. In 83.3% of the patients who experienced a BPS of >5, the RASS value was less than 0.

**Table 1:** RASS at the highest BPS in the pain event group

RASS	−4	−3	−2	−1	0	1	2
Number (percentage)	3 (10.0)	2 (6.7)	9(30.0)	3 (10.0)	8 (27.7)	1 (3.3)	4 (13.3)

Univariate analysis of the differences in patient characteristics is shown in Table 2. Patients in the pain event group had a lower P/F ratio, a lower frequency of fentanyl use, and a higher frequency of inotropic support. Multivariate logistic regression analysis revealed the disuse of fentanyl (odds ratio = 0.35, 95%CI = 0.14–0.89) and the requirement of inotropic support (odds ratio = 4.74, 95%CI = 1.58–12.50) as independent predictors of the development of a BPS of >5 (Table 3). The mean Concordance Index was 0.789 (range, 0.709–0.869).

**Table 2:** Univariate analysis of patient characteristics associated with pain event

	Pain event group	Control group	*p* value
Male sex	21 (70.0)	81 (66.1)	0.686
Age	69.4 ± 10.2	68.2 ± 13.6	0.674
Height (cm)	158.0 ± 8.4	161.0 ± 10.2	0.226
Body Weight (kg)	55.8 ± 15.3	54.1 ± 11.3	0.501
Cardiac surgery	9 (30.0)	43 (35.5)	0.19
Non-cardiac surgery	7 (23.3)	42 (34.7)	
No surgical intervention	14 (46.7)	36 (29.8)	
APACHE II score	21.1 ± 9.51	19.8 ± 8.86	0.488
Blood pressure	109.0 ± 19.2	110.0 ± 19.8	0.883
P/F ratio	211 ± 121	272 ± 118	0.0123
Fentanyl	13 (43.3)	91 (75.2)	<0.001
Inotropic support	24 (80.0)	51 (42.1)	<0.001
Propofol	19 (63.3)	70 (57.9)	0.585
Dexmedetomidine	5 (16.7)	41 (33.9)	0.0666
Midazolam	7 (23.3)	11 (9.09)	0.0312

**Table 3:** Logistic regression analysis for the pain event group

	Odds ratio	95%CI	*p* value
Fentanyl	0.350	0.140-0.890	0.027
Inotropic support	4.440	1.580-12.50	0.005

### Clinical outcomes of elevated BPS during mechanical ventilation

**Fig. 3:**
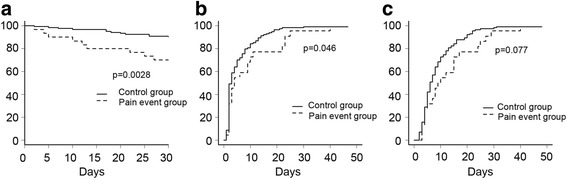
Kaplan-Meier plots showing the relationship between pain event and patient outcome. *Solid lines* demonstrate patients in the control group; *dotted lines* demonstrate patients in the pain episode group. **a** Mortality rate up to 30 days after the initiation of mechanical ventilation. Mortality rate was significantly lower in the control group compared to that in the pain event group (*p* = 0.0028, log-rank test). **b** Time to successful weaning from mechanical ventilation. Time to successful weaning was significantly shorter in the control group compared to that in the pain event group (*p* = 0.046, log-rank test). **c** Length of ICU stay. Length of ICU stay did not differ significantly between the two groups (*p* = 0.077, log-rank test)

Figure 3a demonstrates Kaplan-Meier curves of patient outcomes after the initiation of mechanical ventilation. Thirty-day in-hospital mortality rate was 30.0% in the pain event group and 9.9% in the control group. The mortality rate was significantly higher in the pain event group compared to the control group (Fig. 3a, *p* = 0.003). Multivariable Cox regression analysis demonstrated that the pain event group had a 2.59 times greater risk of death, even after adjusting for APACHE II score, P/F ratio, surgical procedure, and the inotropic support and/or midazolam (Table 4). Median duration until successful weaning from mechanical ventilation was 4 days (2–11) in the pain event group and 3 days (2–7) in the control group. The duration of mechanical ventilation was significantly longer in the pain event group compared to the control group (Fig. 3b, *p* = 0.046). There was a non-significant association between the pain event group and a prolonged time to successful weaning from mechanical ventilation, after adjusting for APACHE II score, P/F ratio, surgical procedure, and the use of propofol, dexmedetomidine, and/or midazolam (Table 4). The median duration of time to ICU discharge was 9 (4–17) days in the pain event group compared to 6 (4–12) days in the control group. Length of ICU stay did not differ between the severe and control groups (Fig. 3c). There was a non-significant association between BPS > 5 condition and duration of time to ICU discharge after adjusting for blood pressure, P/F ratio, surgical procedure, and the use of fentanyl, inotropic agents, propofol, dexmedetomidine, and/or midazolam (Table 4).

**Table 4:** Multivariable analysis of clinical outcomes associated with pain event

	Hazard ratio	95%CI	*p* value
Time to weaning from MV	0.693	0.448–1.072	0.099
Length of ICU stay	0.668	0.420−1.061	0.087
In-hospital mortality	2.590	1.001−6.704	0.049

## Conclusions

We investigated the pain intensity in 151 patients receiving mechanical ventilation using the BPS. The percentage of patients who experienced a pain event, defined as BPS of >5, was 19.9%. The median duration of the period during which the BPS was >5 was 2 h, suggesting that pain event was not maintained for a long period. Inotropic support and fentanyl disuse were identified as independent risk factors for the development of a pain event. Patients who experienced pain event had a higher risk of in-hospital death and longer duration of ICU stay. An association between a pain event and poor clinical outcomes provides further rationale to support the important role of pain assessment and appropriate intervention during mechanical ventilation, as recommended by the PAD guidelines [3].

We limited our observation of the BPS to 48 h after the initiation of mechanical ventilation. This decision was made taking into account the following factors: (1) our preliminary study demonstrated that more than 80% of patients had their highest BPS score recorded within 48 h after receiving mechanical ventilation, (2) early sedation after the initiation of mechanical ventilation is associated with long-term patient outcomes [10], and (3) observation periods exceeding the duration of mechanical ventilation [median = 3 days (2–10)] increases selection bias for the analysis of patient outcomes.

One of the major aims of our study was to document actual pain status during mechanical ventilation. Two evaluation tools, the BPS [4] and critical care pain observation tool (CPOT) [11], are available for evaluating pain intensity in patients who are unable to self-report [5–7]. In our ICU, the BPS is used because the BPS has a validated Japanese language version, but CPOT did not have a validated Japanese version during the observation period of this study, and because the medical staff in our institution are more familiar with the BPS than CPOT. The percentage of patients who experienced BPS > 5 was comparable to previous studies reporting that the incidence of pain during mechanical ventilation varies from 4% [12] to 30% [1]. Together, our results demonstrated that a significant percentage of ventilated patients experience pain at rest. Pain should be treated in a patient who requires critical care [3]. Our results, together with those of previous reports [1, 8, 12, 13], however, demonstrate the difficulty of removing all minor pain from all critically ill patients. Such pain might be associated with such side effects of analgesic treatment as circulatory collapse, respiratory depression, and bowel movement inhibition.

Subgroup analysis of the three components of the BPS showed that patients experiencing a BPS of >5 typically show a mildly tightened or grimacing facial expression, partially bent upper limbs, and mild incompliance with ventilation and coughing. The wide distribution of RASS values during the period in which the pain event demonstrates that an elevated BPS is not always associated with an elevated RASS.

The presence or absence of surgical intervention did not affect the incidence of the development of pain event in our study, which is consistent with the results of a previous study [8]. Patients who require mechanical ventilation must remain immobilized in bed. Furthermore, they require vascular, urethral, and gastric catheters as well as tracheal tube insertion. All of these factors can be sources of pain or discomfort. In addition to the pain originating from mechanical ventilation, disease-associated pain is also common in both surgical and medical patients [8].

Opioids have been widely used for treating pain during mechanical ventilation [14]. We used fentanyl as a first-line opioid for mechanically ventilated patients and did not use other opioids throughout the study. Our findings demonstrated that the disuse of fentanyl increased the risk of increased BPS during mechanical ventilation. This is in accordance with a previous study showing the effect of opioids on the prevention of procedure-related increases in BPS [15], agitation [16], and reduction of pain intensity scores [17] during mechanical ventilation.

We identified inotropic support as another risk factor for the pain event during mechanical ventilation. It is unclear whether the inotropic support has a direct effect on pain scores, or whether patient conditions requiring inotropic support are associated with a higher BPS. The correlation between inotropic support and disuse of fentanyl in this study was low (*r* = 0.16). An experimental animal study revealed that a higher blood catecholamine concentration is associated with increased pain sensitivity via the activation of β-adrenergic receptors in the peripheral sensory nerves [18].

Increased BPS occurring early after the initiation of mechanical ventilation was associated with lower survival rate. Pain increases sympathetic tone and evokes a stress response in ventilated patients [3]. Tachycardia, increased myocardial oxygen consumption [19], hypercoagulability [20], immunosuppression [21], and catabolism [22], are all associated with pain in critically ill patients and might partly explain the poor prognosis in patients who experienced a BPS of >5.

In our study, patients with pain event tended to be required longer duration of mechanical ventilation. Inadequate pain control is known to reduce patient-ventilator synchrony [23]. Patient-ventilator asynchrony may be a cause of ventilator-associated lung injury and may negatively affect prognosis [24, 25]. Severe pain is also associated with the development of agitation [26]. Agitation in ventilated patients negatively affects outcomes [27].

To the best of our knowledge, this is the first report describing an association between elevated BPS during mechanical ventilation and poor clinical outcomes. It may be worthwhile to investigate, therefore, whether analgesic intervention to prevent elevation of the BPS during mechanical ventilation can improve patient outcomes in a future prospective study.

There are several limitations to this study. This study was conducted in a retrospective manner and may have missed relevant clinically important confounders. Also, the study was conducted in a single ICU in a tertiary referral hospital which may have influenced the study sample. Furthermore, we used BPS instead of a subjective pain scale (such as the numerical rating scale or visual analog scale). Enrolled patients were not limited to those who were diagnosed as ARDS. Furthermore, since we did not routinely use neuromuscular blockade in these patients, those with severe ARDS could not be excluded from this study. Finally, we were not able to determine the exact reason for death.

In conclusion, we found that 20% of ventilated patients experienced a BPS of >5. An elevated BPS was associated with inotropic support and disuse of fentanyl, and was also associated with poor patient outcomes.
